# Supplementary material for: Pet Owners’ Knowledge of Antibiotic Use and Antimicrobial Resistance and Their Antibiotic Practices: Comparison Between Contexts of Self and Pet
Source: Antibiotics (Basel). 2025 Feb 5;14(2):158. doi: 10.3390/antibiotics14020158 (PMC11851957; doi:10.3390/antibiotics14020158)
Supplement: Supplementary file 1 [file antibiotics-14-00158-s001.zip › Supplementary materials S1- Pet Survey Questionnaire.pdf]

Questionnaire

Thank you for participating in our survey. This questionnaire consists of 3 related sections, with each section focusing on different aspects of antibiotics and antibiotic resistance.

Q1. How long have you been a cat/dog owner? __________________years__________________months

Q2. How many pet cats/dogs have you ever owned?__________________

Q3. How many pet cats/dogs do you currently own?

Cats: __________________

Dogs: __________________

Section A

This section seeks your views on antibiotic use and antibiotic resistance for your pet cat or dog.

*Please **TICK** the response category that applies to you.*

Use of Antibiotics

Q4. Has your pet cat/dog ever taken antibiotics (oral, intramuscular [injection], intravenous [drip], topical [cream/ointment/lotion/ear or eye drops] etc.)?

	Please tick ONE response
Yes	☐ 1
No (Skip to Q9)	☐ 2
Don't know whether my pet has ever taken antibiotics, for e.g. the seller or groomer might have given my pet an antibiotic without my knowledge (Skip to Q9)	☐ 3

Q5. When did your pet cat/dog last take antibiotics (oral, intramuscular [injection], intravenous [drip], topical [cream/ointment/lotion/ear or eye drops] etc.)?

	Please tick ONE response
In the last month	☐ 1
In the last 6 months	☐ 2
In the last year	☐ 3
More than a year ago	☐ 4
Cannot remember when	☐ 5

Q6. What form(s) of antibiotics has your pet cat/dog ever taken?

	Please tick ALL that applies
Tablets/capsules	☐ 1

Oral liquid	☐ 2
Injection	☐ 3
Intravenous (IV) drip	☐ 4
Topical e.g. cream/ointment/lotion/ear drops/eye drops	☐ 5
Others (Please specify): ________________________	☐ 6
Administration route not known / Unsure	☐ 7

Q7. Have you ever gotten advice from a veterinary practitioner (vet), veterinary technician or veterinary nurse on how to administer the antibiotic(s) for your pet cat/dog?

Please tick ONE response

Yes, I have received advice on how to administer the antibiotic(s) (e.g. for how many times a day, for how many days)	☐ 1
No	☐ 2
Cannot remember	☐ 98

Q8. On the scale shown, how much do you agree with the following statements on antibiotic use for your pet cat/dog?

	**Strongly Disagree**	**Disagree**	**Neither Agree Nor Disagree**	**Agree**	**Strongly Agree**
a) I normally keep antibiotic stocks for my pet at home in case of emergency	☐1	☐2	☐ 3	☐ 4	☐ 5
b) If my pet is sick, I will usually give my antibiotics to it	☐1	☐2	☐ 3	☐ 4	☐ 5
c) I will save leftover antibiotics for future use for my pet	☐1	☐2	☐ 3	☐ 4	☐ 5
d) I normally stop giving my pet antibiotics when it starts feeling better or when symptom(s) subside	☐1	☐2	☐ 3	☐ 4	☐ 5
e) I will see another veterinary practitioner (vet) if my pet's vet does not give it antibiotics	☐1	☐2	☐ 3	☐ 4	☐ 5
f) I will give my pet leftover antibiotics when I think it needs them	☐1	☐2	☐ 3	☐ 4	☐ 5

Knowledge of Antibiotic Use for Pet Cats and Dogs

Q9. When do you think you should stop giving antibiotics to your pet cat/dog once it has begun treatment?

Please tick ONE response

When it feels better or when symptom(s) subside	☐ 1
When it has taken all of the antibiotics as directed	☐ 2
Don't know	☐ 99

Q10. Please indicate whether you think the following statements are 'True' or 'False'.

	True	False	Don't Know
a) Antibiotics can treat bacterial infections in pet cats/dogs	☐ 1	☐ 2	☐ 99
b) Antibiotics can treat viral infections in pet cats/dogs	☐ 1	☐ 2	☐ 99
c) Antibiotics should always be prescribed for respiratory tract infections for pet cats/dogs	☐ 1	☐ 2	☐ 99
d) Pet cats/dogs need antibiotics after a routine sterilization or desexing surgery	☐ 1	☐ 2	☐ 99
e) An abscess (i.e. a tissue cavity filled with pus) from a bite wound will usually heal without antibiotics	☐ 1	☐ 2	☐ 99
f) Antibiotics do not have side effects in pet cats/dogs	☐ 1	☐ 2	☐ 99
g) Bacteria can become resistant to antibiotics used in pet cats/dogs	☐ 1	☐ 2	☐ 99
h) The more antibiotics we use in society, the higher the risk that antibiotic resistance develops	☐ 1	☐ 2	☐ 99
i) The use of antibiotics in pet cats/dogs can reduce the effectiveness of antibiotics in humans	☐ 1	☐ 2	☐ 99
j) It is okay to use antibiotics for my pet cat/dog that were given to another pet cat/dog, as long as they were being used to treat the same illness.	☐ 1	☐ 2	☐ 99
k) It is okay to buy the same antibiotics or request them from a veterinary practitioner (vet), if they helped my pet cat/dog get better previously when it had the same symptoms	☐ 1	☐ 2	☐ 99

Understanding of Antimicrobial Resistance in Pet Cats and Dogs

Q11. Please indicate whether you think the following statements are 'True' or 'False'.

	True	False	Don't Know
a) Antibiotic resistance occurs when a pet cat/dog's body becomes resistant to antibiotics and they no longer work as well	☐ 1	☐ 2	☐ 99
b) Many infections in pet cats/dogs are becoming increasingly resistant to treatment by antibiotics	☐ 1	☐ 2	☐ 99
c) If bacteria are resistant to antibiotics, it can be very difficult or impossible to treat the infections they cause in pet cats/dogs	☐ 1	☐ 2	☐ 99
d) Antibiotic resistance in pet cats/dogs is an issue that could affect me or my family	☐ 1	☐ 2	☐ 99
e) Antibiotic resistance in pet cats/dogs is an issue in other countries but not here	☐ 1	☐ 2	☐ 99
f) Antibiotic resistance is only a problem for pet cats/dogs which take antibiotics regularly	☐ 1	☐ 2	☐ 99
g) Bacteria which are resistant to antibiotics can be spread from pet cats/dogs to their owners	☐ 1	☐ 2	☐ 99

h) Bacteria which are resistant to antibiotics can be spread from owners to their pet cats/dogs	☐ 1	☐ 2	☐ 99
i) Antibiotic-resistant infections could make medical procedures like surgery much more dangerous for pet cats/dogs	☐ 1	☐ 2	☐ 99

Section B

This section seeks your views on antibiotic use and antibiotic resistance for yourself.
*Please **TICK** the response category that applies to you.*

Use of Antibiotics in Humans

Q12. On the scale shown, how much do you agree with the following statements on antibiotic use for yourself?

	Strongly Disagree	Disagree	Neither Agree Nor Disagree	Agree	Strongly Agree
a) I normally keep antibiotic stocks at home in case of emergency	☐1	☐2	☐ 3	☐ 4	☐ 5
b) If my family member is sick, I will usually give my antibiotics to them	☐1	☐2	☐ 3	☐ 4	☐ 5
c) I will save leftover antibiotics for future use	☐1	☐2	☐ 3	☐ 4	☐ 5
d) I normally stop taking antibiotics when I start feeling better	☐1	☐2	☐ 3	☐ 4	☐ 5
e) I will see another doctor if my doctor does not give me antibiotics	☐1	☐2	☐ 3	☐ 4	☐ 5
f) I will take leftover antibiotics when I think I need them	☐1	☐2	☐ 3	☐ 4	☐ 5

Knowledge of Antibiotic Use in Humans

Q13. When do you think you should stop taking antibiotics once you've begun treatment?

	Please tick ONE response
When you feel better	☐ 1
When you've taken all of the antibiotics as directed	☐ 2
Don't know	☐ 99

Q14. Please indicate whether you think the following statements are 'True' or 'False'.

	True	False	Don't Know
a) It's okay to use antibiotics that were given to a friend or family member, as long as they were used to treat the same illness	☐ 1	☐ 2	☐ 99
b) It's okay to buy the same antibiotics or request for them from a doctor, if they had helped you get better previously when you had the same symptoms.	☐ 1	☐ 2	☐ 99

Understanding of Antimicrobial Resistance in Humans

Q15. Please indicate whether you think the following statements are 'True' or 'False'.

	True	False	Don't Know
a) Antibiotic resistance occurs when your body becomes resistant to antibiotics and they no longer work as well	☐ 1	☐ 2	☐ 99
b) Many infections are becoming increasingly resistant to treatment by antibiotics	☐ 1	☐ 2	☐ 99
c) If bacteria are resistant to antibiotics, it can be very difficult or impossible to treat the infections they cause	☐ 1	☐ 2	☐ 99
d) Antibiotic resistance is an issue that could affect me or my family	☐ 1	☐ 2	☐ 99
e) Antibiotic resistance is an issue in other countries but not here	☐ 1	☐ 2	☐ 99
f) Antibiotic resistance is only a problem for people who take antibiotics regularly	☐ 1	☐ 2	☐ 99
g) Bacteria which are resistant to antibiotics can be spread from person to person	☐ 1	☐ 2	☐ 99
h) Antibiotic-resistant infections could make medical procedures like surgery, organ transplants and cancer treatment much more dangerous	☐ 1	☐ 2	☐ 99

Section C

We need to find out a little about your background to help us better understand the information you shared with us. All detail in this questionnaire will be kept strictly confidential.
*Please **TICK** the response category that applies to you.*

Q18. What is your residency status?

	Please tick ONE response
Singapore Citizen	☐ 1
Permanent Resident (PR)	☐ 2
Foreigner	☐ 3

Q19. What is your birth year? ____________________

Q20. What is your ethnicity?

	Please tick ONE response
Chinese	☐ 1
Malay	☐ 2
Indian	☐ 3
Others	☐ 4

Q21. What is your gender?

	Please tick ONE response
Female	☐ 1
Male	☐ 2

Q22. What is your current marital status?

	**Please tick <u>ONE</u> response**
Never married	☐ 1
Married	☐ 2
Separated	☐ 3
Divorced	☐ 4
Widowed	☐ 5

Q23. What is your current work status?

	**Please tick <u>ONE</u> response**
Full-time work	☐ 1
Part-time work	☐ 2
Looking for work	☐ 3
Retired	☐ 4
Homemaker	☐ 5
Unemployed (able to work)	☐ 6
Unemployed (unable to work due to medical conditions or disabilities)	☐ 7
Student	☐ 8
Others (Please specify): ________________________	☐ 9

Q24. What is your highest educational qualification?

	**Please tick <u>ONE</u> response**
Below Secondary	☐ 1
Secondary	☐ 2
Post-Secondary	☐ 3
Diploma and Professional Qualification	☐ 4
University	☐ 5
Post-Graduate Degree	☐ 6

Q25. Please select the fields in which you have ever undertaken any study/and or work.

	**Please tick <u>ALL</u> that applies**
Human health	☐ 1
Animal health	☐ 2
Agriculture or animal production	☐ 3
Scientific research	☐ 4
Scientific education	☐ 5
NONE of the above	☐ 6

Q26. Which best describes your current residence?

	Please tick **ONE** response
HDB 1- & 2-room flats	☐ 1
HDB 3-room flats	☐ 2
HDB 4-room flats	☐ 3
HDB 5-room & Executive flats/condominiums	☐ 4
Condominiums & Other apartments	☐ 5
Landed properties	☐ 6
Others (Please specify): ________________________	☐ 7

This is the end of the questionnaire. Thank you very much for your time and kind cooperation. We have a small token of appreciation for you, upon collection of your completed questionnaire.

Note: Only questions relevant to the study are shown
