# Supplementary material for: Pet Owners’ Knowledge of Antibiotic Use and Antimicrobial Resistance and Their Antibiotic Practices: Comparison Between Contexts of Self and Pet
Source: Antibiotics (Basel). 2025 Feb 5;14(2):158. doi: 10.3390/antibiotics14020158 (PMC11851957; doi:10.3390/antibiotics14020158)
Supplement: Supplementary file 1 [file antibiotics-14-00158-s001.zip › Supplementary materials S2 (Figures and Table).pdf]

Supplementary materials

Figure S1 - Knowledge of antibiotic use for pets among 1080 pet owners of dogs and/or cats overall and by type of pet owned

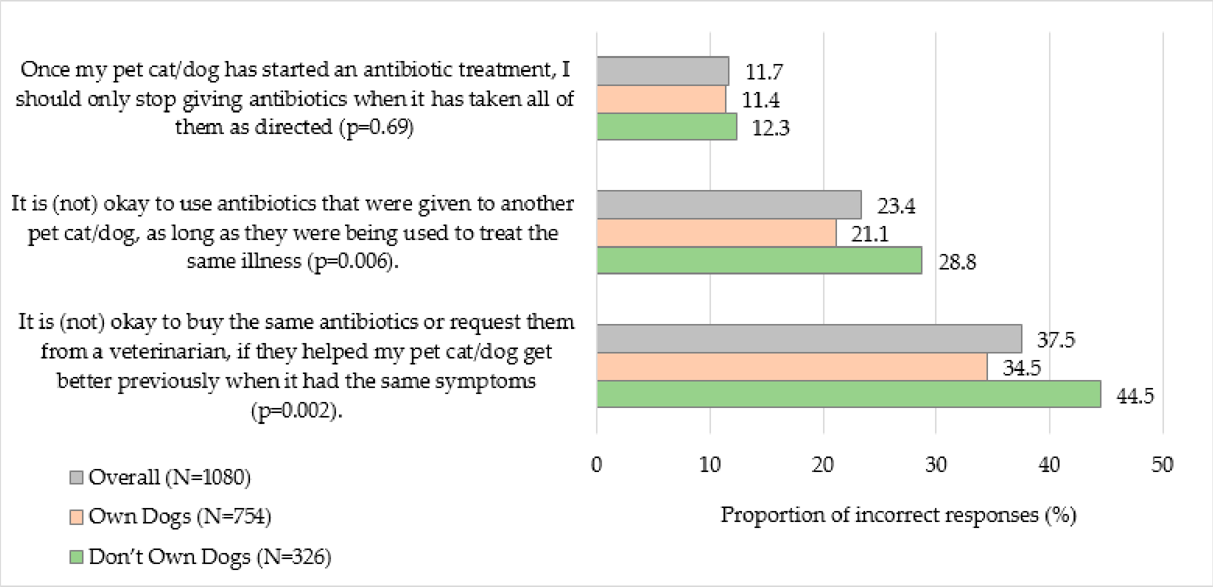

*P value based on Pearson Chi square

Table S1 – Additional statements on knowledge of AMR in humans and in pets among 1080 pet owners of dogs and/or cats

**In humans**		**In pets**		**P value***
**AMR Statements**	**Proportion of correct responses**	**AMR Statements**	**Proportion of correct responses**	
If bacteria are resistant to antibiotics, it can be very difficult to treat the infections they cause (True)	72.5%	If bacteria are resistant to antibiotics, it can be very difficult to treat the infections they cause in pet cats/dogs (True)	69.6%	**0.03**
Antibiotic-resistant infections could make medical procedures much more dangerous (True)	60.4%	Antibiotic-resistant infections could make medical procedures much more dangerous for pet cats/dogs (True)	58.2%	0.08
Antibiotic resistance is only a problem for people who take antibiotics regularly (False)	37.0%	Antibiotic resistance is only a problem for pet cats/dogs which take antibiotics regularly (False)	35.3%	0.22

*P values based on McNemar Chi Square test

Figure S2 – Knowledge of antimicrobial resistance (AMR) in pets among the 1080 pet owners of dogs and/or cats overall and by type of pet owned

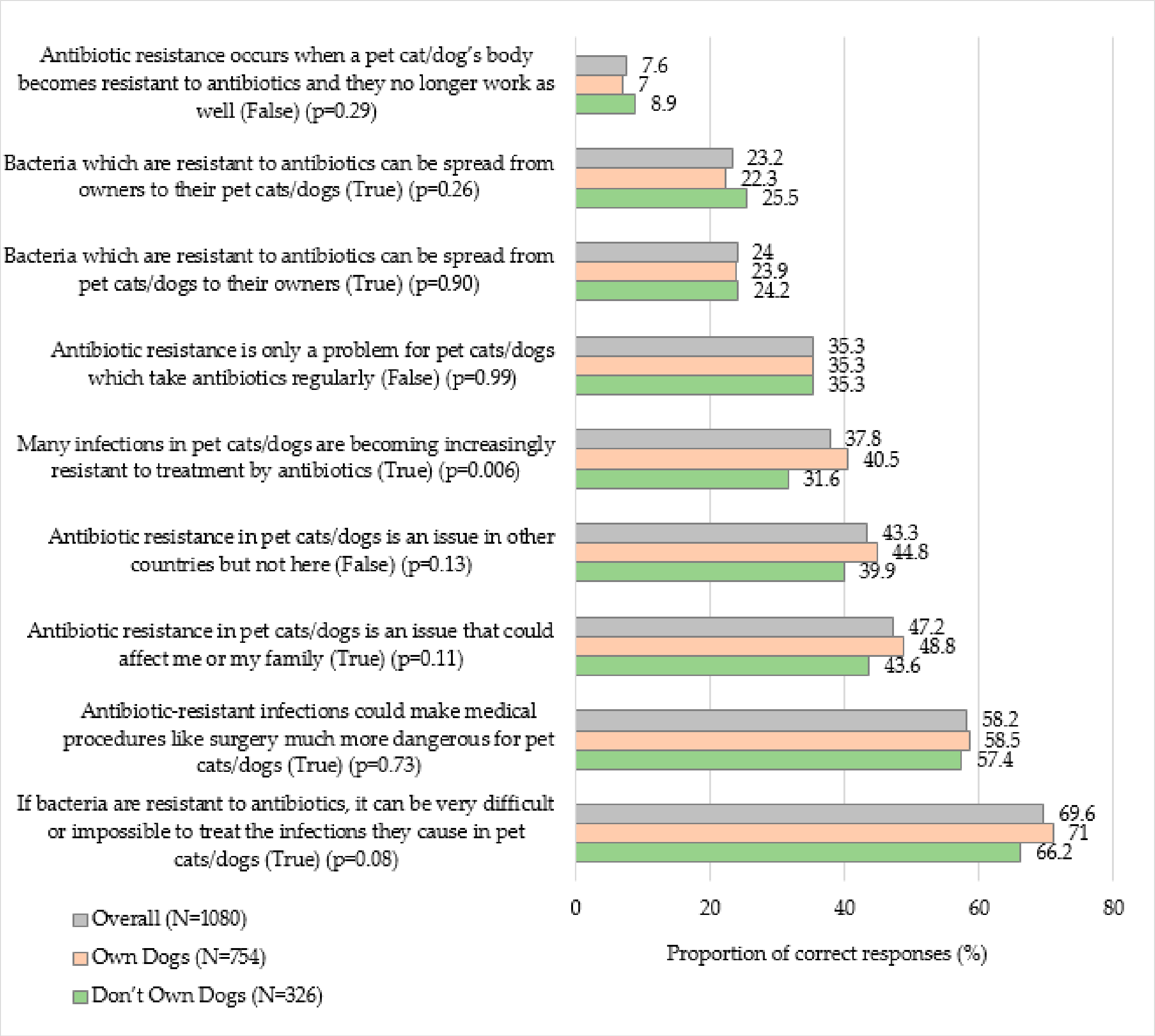

*P value based on Pearson Chi square

Figure S3 - Antibiotic use practices for pets among 1080 pet owners of dogs and/or cats overall and by type of pet owned

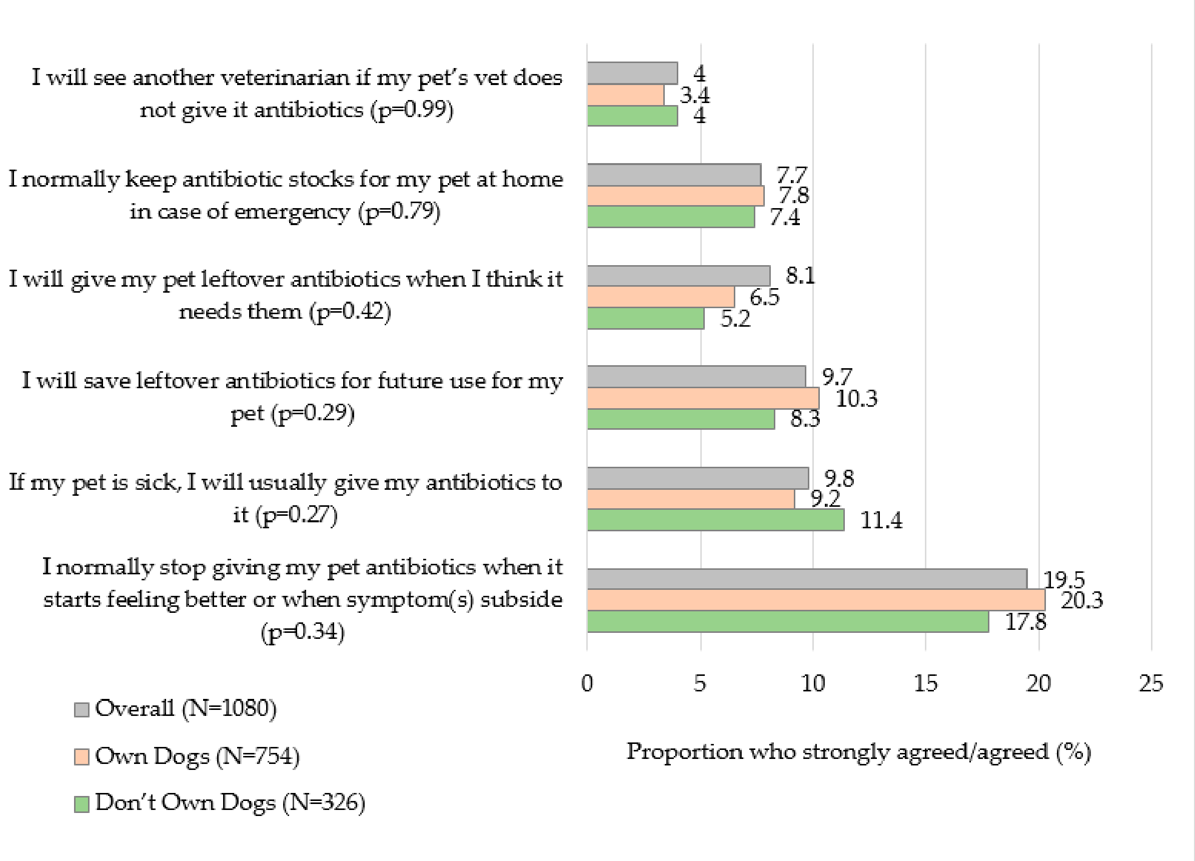

*P value based on Pearson Chi square test
